# An Association Rule Mining-Based Framework for Understanding Lifestyle Risk Behaviors

**DOI:** 10.1371/journal.pone.0088859

**Published:** 2014-02-13

**Authors:** So Hyun Park, Shin Yi Jang, Ho Kim, Seung Wook Lee

**Affiliations:** 1 Graduate School of Public Health, Seoul National University, Seoul, Korea; 2 Cardiovascular Imaging Center, Samsung Medical Center, Seoul, Korea; National Taiwan University, Taiwan

## Abstract

**Objectives:**

This study investigated the prevalence and patterns of lifestyle risk behaviors in Korean adults.

**Methods:**

We utilized data from the Fourth Korea National Health and Nutrition Examination Survey for 14,833 adults (>20 years of age). We used association rule mining to analyze patterns of lifestyle risk behaviors by characterizing non-adherence to public health recommendations related to the Alameda 7 health behaviors. The study variables were current smoking, heavy drinking, physical inactivity, obesity, inadequate sleep, breakfast skipping, and frequent snacking.

**Results:**

Approximately 72% of Korean adults exhibited two or more lifestyle risk behaviors. Among women, current smoking, obesity, and breakfast skipping were associated with inadequate sleep. Among men, breakfast skipping with additional risk behaviors such as physical inactivity, obesity, and inadequate sleep was associated with current smoking. Current smoking with additional risk behaviors such as inadequate sleep or breakfast skipping was associated with physical inactivity.

**Conclusion:**

Lifestyle risk behaviors are intercorrelated in Korea. Information on patterns of lifestyle risk behaviors could assist in planning interventions targeted at multiple behaviors simultaneously.

## Introduction

Lifestyle risk behaviors such as smoking, excessive alcohol consumption, obesity, and physical inactivity are known to increase the risk of chronic diseases and mortality [1] and contribute to 31.3% of the cost of illness in Korea [2]. Lifestyle risk behaviors pose a major public health concern and have therefore been targeted for behavioral change.

Individuals often exhibit multiple lifestyle risk behaviors; therefore, it is important to study individuals who exhibit more than one lifestyle risk behavior. Indeed, the presence of two or more risk behaviors is associated with an increased risk of cardiovascular disease [3], cancer [4], and mortality [5], and such risk is greater than would be expected for the sum of the separate behavioral effects. Knowledge of patterns of risk behaviors can be useful for developing prevention strategies. An understanding of risk behaviors can aid in the discrimination of subgroups with risky patterns so that prevention programs can be better targeted and organized [6].

A number of previous studies have examined multiple lifestyle risk behaviors of different types and quantities. In the US population, 17% reported three or more risk behaviors [7]. In Hong Kong, about 5% of older adults reported at least 3 risk behaviors [8], and among Korean male adults, about 15% reported three risk behaviors [9]. These studies examined smoking, excessive alcohol consumption, physical inactivity, and inadequate diet. Maintaining proper weight, sleeping sufficiently at night, eating breakfast, and avoiding frequent snacking have been proposed as additional public health recommendations; however, little research has investigated these behavioral patterns, that is, the associations of three or more lifestyle risk behaviors. These behaviors as they appear in our daily lives are more comprehensive than previous research has indicated.

To this end, the present study aimed to investigate the prevalence and patterns of lifestyle risk behaviors among Korean adults using association rule mining (ARM). The current study is the first of its type to focus on patterns of three or more lifestyle risk behaviors simultaneously.

## Methods

### Data

We utilized data from the Fourth Korea National Health and Nutrition Examination Survey (KNHANES), a survey conducted by the Division of Chronic Disease Surveillance [10]. The KNHANES is a cross-sectional, nationwide, population-based survey that has been conducted periodically since 1998 to assess the health and nutritional status of the South Korean population [10]. KNHANES IV data (available at https://knhanes.cdc.go.kr) were collected between 2007 and 2009. We analyzed data from 14,833 individuals (5,908 men, 8,925 women, all >20 years of age) who had completed health behavior questionnaires. The present study received ethical approval from the Institutional Review Board of the Graduate School of Public Health at Seoul National University (IRB No: 12-2013-04-03) in Seoul, Korea.

### Definition of Variables

We considered lifestyle risk behaviors in terms of non-adherence to the Alameda 7 health behaviors criteria. The Alameda 7 health behaviors have been studied since the 1960 s in Alameda county, California (US) to investigate the relationship between health status and long-term survival [11]. Risk behaviors included current smoking (CS), heavy drinking of alcoholic beverages (HD), physical inactivity (PI), obesity (OB), inadequate sleep (IS), breakfast skipping (BS), and frequent snacking (FS). To investigate the patterns of lifestyle risk behaviors and compare to previous studies, all health behavior variables were dichotomized, and risk behaviors were determined by the following criteria:

- CS was defined as currently smoking whereas non-smoking was defined as either formerly smoking or never smoking [7], [12].
- HD was assessed by the quantity/frequency questionnaire items and estimates, and defined in terms of the number of grams of alcohol consumed daily. HD was defined as the consumption of 25 g/day or more of alcohol [13], [14]. Participants with alcohol consumption below this level were not included in the heavy drinking group.
- PI was defined as the lack of participation in either moderate-intensity aerobic physical activity (for a minimum of 30 min, 5 days per week) or vigorous-intensity aerobic activity (for a minimum of 20 min, 3 days per week) [15]. Participants with activity levels greater than these cut-off levels were considered physically active.
- OB was defined by a BMI ≥25 kg/m^2^, whereas non-obesity was defined by a BMI <25 kg/m^2^ [16]. We used the obesity variable (based on BMI score) to assess the maintenance of healthy weight.
- IS was defined as sleeping either less than 7 or more than 8 h per night, whereas adequate sleep was defined as sleeping between 7 and 8 h per night [17].
- BS was defined as not eating breakfast either that day or the day before, whereas breakfast eating was defined as having eaten breakfast that day.
- FS was defined as eating between meals 3 or more times per day, whereas infrequent snacking as fewer than 3 times per day, regardless of type and quantity.

The analysis incorporated 6 sociodemographic variables: sex, age, marital status, education, occupation, and income. The age variable was divided into three categories: (1) 20–44 years, (2) 45–64 years, and (3) ≥65 years. Marital status was categorized as (1) married, (2) separated/divorced/widowed, and (3) never married. Education was categorized as follows: (1) either elementary school or no school at all, (2) middle school, (3) high school, and (4) college and above. Occupation was categorized as (1) office work, (2) manual work, and (3) unemployed. Income was measured by equivalent income based on the number of family members [18], and was categorized in quartiles.

In addition to the lifestyle risk variables, we incorporated personal health variables in the analysis. Perceived stress was categorized according to yes or no responses, and participants’ self-rated health status was categorized as either good/fair or bad. We also included chronic disease variables, in accordance with previous research demonstrating that health behaviors affect health status. The diseases included were cancer [14], [19], hypertension [13], coronary artery disease [19], cerebrovascular attack [19], chronic obstructive pulmonary disease [19], [20], diabetes [20], dyslipidemia [20], gastric ulcers [19], liver disease [13], back pain [21], osteoporosis [22], and depressive disorders [23]. The presence of each disease was confirmed if respondents had been diagnosed by a doctor. All versions of the KNHANES surveyed these diseases.

### Comparison between ARM and Other Methods

To assess the co-occurrence of behavioral variables in a dataset wherein variables are treated equally, we consider ARM a more suitable method as opposed to regression modeling. Although regression modeling allows for the testing of statistical interactions among independent variables and assesses differences in the effects of one or more independent variables across levels of another independent variable [24], in cases where it is used as a method for selecting variables, it is difficult to interpret the meaning of the variable combinations [25]. The tree structure is the recommended system when the order of the manifesting variables is of substantial importance, but it is not suitable for evaluating simple combinations [25]. For these reasons, we selected ARM as the primary method in the current study.

### Association Rule Mining (ARM)

ARM, also known as market basket analysis (MBA), is a popular data mining method designed to identify groups of variables that are highly correlated with each other, or with respect to a specific target variable [26]. The strength of this method of analysis is that ARM measures the support, confidence, and lift of the rule, as explained below.

The support for the rule (A⇒B) is the probability that the two behaviors occur together.
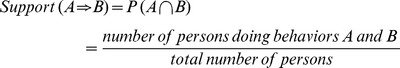



The confidence of an association rule (A⇒B) is the conditional probability of B behavior given that a person performs behavior A.
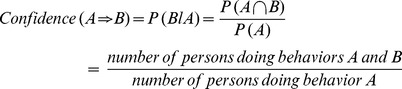



The lift of the rule (A⇒B) is the confidence of the rule divided by the *expected* confidence, assuming that the behaviors are independent. The lift of the rule, then, is the confidence divided by the support.
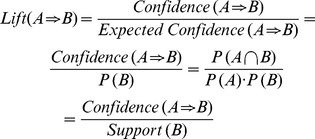



The lift is interpreted as a general measure of the association between the behavior sets. Values greater than 1 indicate a positive correlation, values equal to 1 indicate zero correlation, and values less than 1 indicate a negative correlation.

ARM is used in many fields of study, including not only market research, but also medicine and epidemiology. For example, ARM has been applied in areas such as the prediction of acute myocardial infarction [27], studies of ADHD comorbidity [28], and cancer prevention factors [29].

### Statistical Analysis

**Figure 1:**
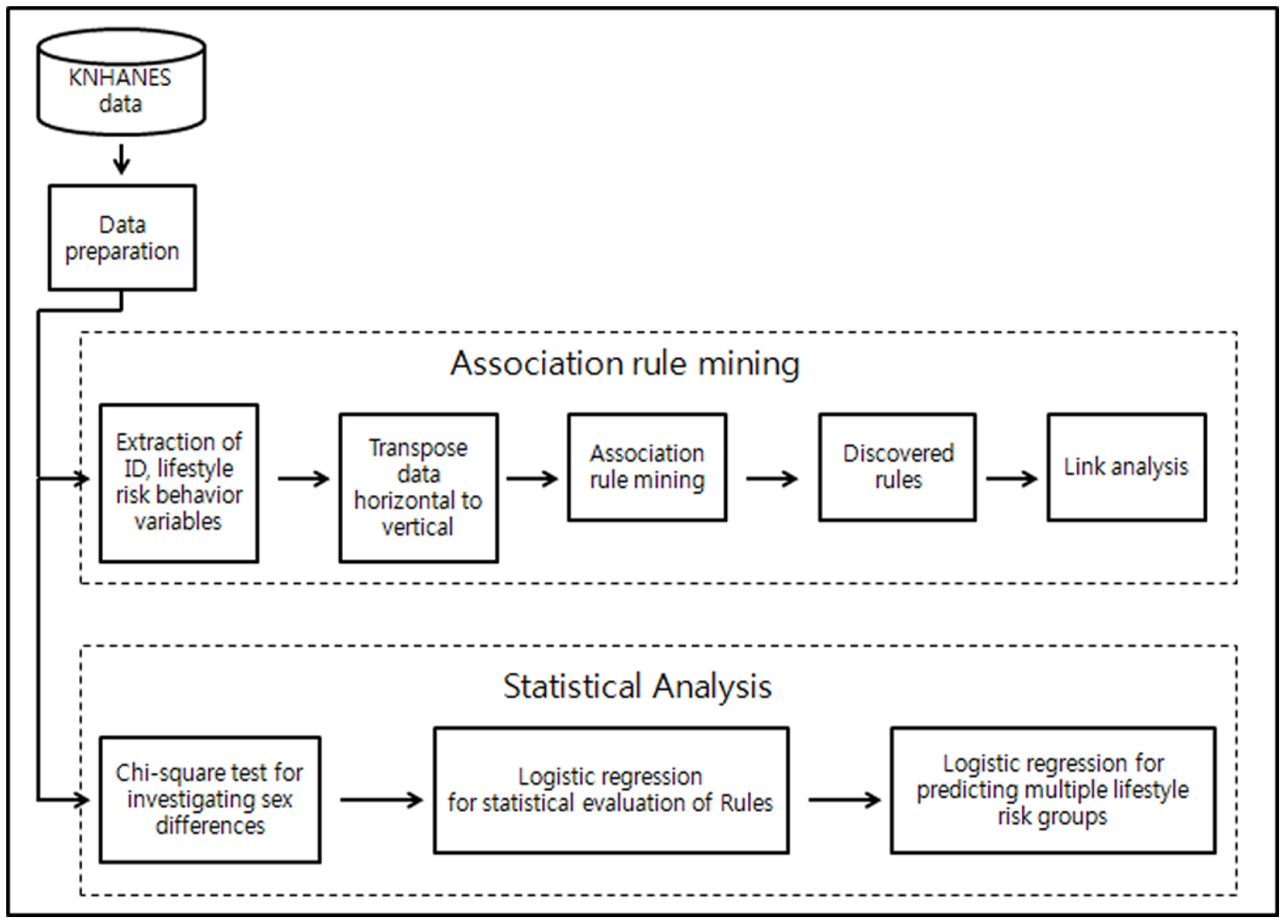
Analysis framework.

Figure 1 illustrates the framework of the study analysis. We utilized association rule mining (ARM) to determine the associations among the lifestyle risk behaviors. To avoid redundant rules, we established a support threshold of 2%, and as there were fewer rules for women than men, we used confidence thresholds of 50% for women and 60% for men. Among women, there were no rules at the 60% confidence levels; among men, there were 19 similar rules at the 50% confidence level. Thus, we set the confidence threshold differently. A support threshold of 2% meant that we accepted the rule only if there was an observed frequency of 2% or greater for the possible combination of behaviors. Our sample size was 5,908 men and 8,925 women, and we believed that 2% of support could improve statistical inference. Further, a confidence threshold of 50% meant that the conditional probability of the co-occurrence of two variables was 50% or greater. We set the threshold of lift as “over 1.” A lift threshold over 1 meant that we accepted the positive association rule.

To examine the differences between the male and female groups, chi-square tests were conducted on the seven health risk behaviors (CS, HD, PI, OB, IS, BS, and FS). We calculated the number of participants’ risk behaviors from 0 (none of the behaviors) to 7 (all of the behaviors). We used sample weights to calculate the prevalence of behaviors to test for sex differences, and to conduct a multiple logistic regression analysis.

After determining the association rules, we conducted a multiple logistic regression analysis to evaluate the lift value according to the odds ratio (with a 95% confidence interval), and to predict multiple behavior patterns. We used SAS® version 9.3 and SAS Enterprise Miner® version 4.3 for the ARM analysis and to construct the model figure [28].

## Results

### Prevalence of Lifestyle Risk Behaviors

Table 1 presents the characteristics of the study participants. There were observed sex differences in age distribution, marital status, education, occupation, income, and chronic disease status. Men were more likely to have chronic diseases (*p*<0.001) and to report more lifestyle risk behaviors (*p*<0.001). The proportion of participants who reported no risk behaviors was 5.4% for women and 3.6% for men. No participants reported all 7 risk behaviors. Approximately 72% of the sample reported 2 or more lifestyle risk behaviors.

**Table 1:** Characteristics of the study population.

	Weighted N(%)^a^	Women %	Men %	*p*-value
Age				<.0001
20–44	19,131,622(52.8)	50.7	54.9	
45–64	12,177,541(33.6)	33.3	33.9	
65+	4,931,074(13.6)	16.0	11.2	
Marital status				<.0001
Married	25,287,699(70.1)	68.2	72.0	
Separated/Divorced/Widowed	4,002,312(11.1)	17.3	4.8	
Never married	6,801,593(18.8)	14.5	23.2	
Education				<.0001
College, University	10,691,450(29.5)	25.3	33.8	
High school	14,402,050(39.8)	37.7	41.9	
Middle school	3,835,570(10.6)	10.5	10.7	
No or elementary school	7,273,591(20.1)	26.5	13.6	
Occupation				<.0001
Office worker	2,940,620(9.4)	7.1	12.1	
Manual worker	13,957,802(44.8)	32.1	59.0	
Unemployed	14,253,983(45.8)	60.8	29.0	
Income level				<.0001
1^st^ quartile	10,669,193(30.2)	29.2	31.3	
2^nd^ quartile	10,257,925(29.1)	28.4	29.8	
3^rd^ quartile	8,763,902(24.8)	25.4	24.3	
4^th^ quartile	5,603,085(15.9)	17.1	14.7	
Number of lifestyle risk behaviors				<.0001
0	1,646,405(4.5)	5.4	3.6	
1	8,283,253(22.9)	28.6	17.0	
2	12,594,732(34.8)	38.2	31.2	
3	8,740,052(24.1)	21.3	26.9	
4	3,744,086(10.3)	5.4	15.4	
5	1,082,057(3.0)	0.9	5.1	
6	149,652(0.4)	0.2	0.6	
7	0(0.0)	0	0	
Chronic disease^b^				<.0001
No	16,714,065(46.1)	50.4	41.8	
Yes	19,526,172(53.9)	49.6	58.2	

a Proportions were calculated by a survey frequency procedure using sample weights from the survey.

b Chronic disease includes cancer, hypertension, coronary artery disease, cerebrovascular attack, chronic obstructive pulmonary disease, diabetes, dyslipidemia, gastric ulcer, liver disease, back pain, osteoporosis, and depressive disorder.

**Figure 2:**
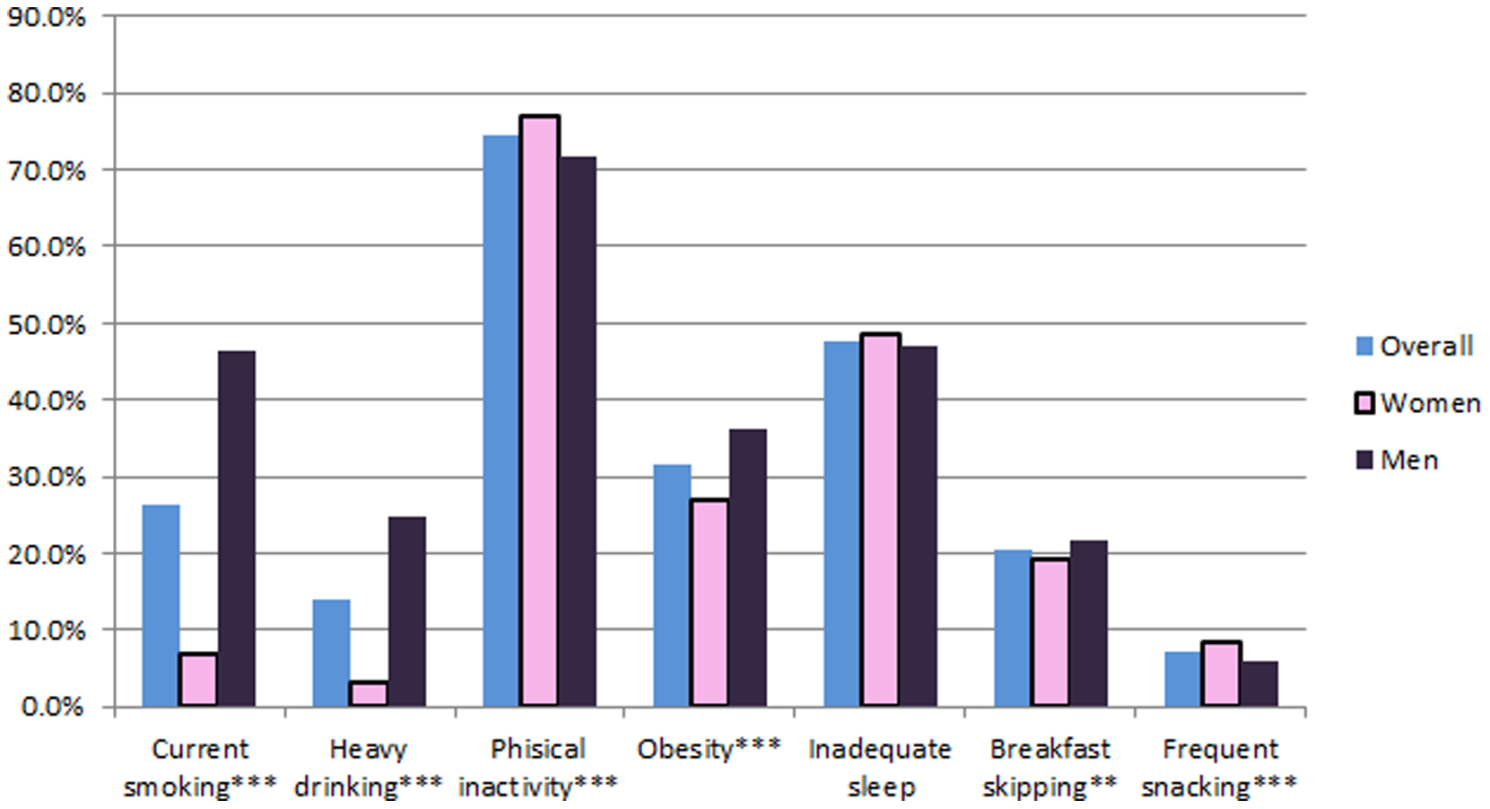
Estimated prevalence of lifestyle risk behaviors in adults aged ≥20 years. *p<.05, **p<.01, ***p<.001.

The prevalence of individual lifestyle risk behaviors is shown in Figure 2. According to our criteria, the majority of participants were physically inactive (77.0% of women and 71.8% of men). Approximately 48% of participants reported sleeping for an inadequate amount of time. Men were more likely to smoke and drink alcohol heavily (*p*<0.001), while women were more likely to be physically inactive (*p*<0.001).

### The ARM Analyses of Lifestyle Risk Behaviors

Table 2 shows the results of the ARM analysis. We show the left side of the discovered rules equations as predictors, and the right side of the rules equations as the predicted variables. In addition, we present lift values from ARM and adjusted odds ratios with their 95% CIs from the logistic regression analysis to statistically evaluate the ARM results. The majority of ARM results were significant at the.05 level.

**Table 2:** Results of association rule mining^a^ and multiple logistic regression analysis of lifestyle risk behaviors.

Size^b^	Predictors	Predicted	Support(%)	Confidence(%)	Lift	OR^c^ (95% CI)
Total	(n = 14,833)					
3	PI & OB	IS	12.51	51.84	1.00	1.08 (1.00, 1.17)
2	HD	CS	4.63	55.64	2.61	2.44 (2.13, 2.80)
3	PI & HD	CS	3.13	56.41	2.64	2.36 (2.01, 2.77)
3	IS & HD	CS	2.30	55.03	2.58	2.22 (1.85, 2.67)
Women	(n = 8,925)					
2	OB	IS	16.82	53.45	1.02	1.07 (0.97, 1.18)
3	PI & OB	IS	12.71	54.14	1.04	1.08 (0.98, 1.20)
2	**CS**	**IS**	**3.40**	**55.34**	**1.06**	**1.27 (1.05, 1.52)**
3	PI & CS	IS	2.62	55.14	1.06	1.22 (0.99, 1.50)
3	OB & BS	IS	2.23	53.13	1.02	1.27 (1.02, 1.58)
Men	(n = 5,908)					
3	PI & BS	CS	7.17	60.51	1.38	1.73 (1.45, 2.05)
3	IS & BS	CS	4.66	61.54	1.40	1.76 (1.43, 2.17)
3	OB & BS	CS	3.80	62.32	1.42	1.77 (1.40, 2.23)
4	PI & IS & BS	CS	3.59	64.86	1.48	2.06 (1.61, 2.63)
4	PI & OB & BS	CS	2.86	65.59	1.50	2.07 (1.57, 2.73)
3	**HD & BS**	**CS**	**2.24**	**66.84**	**1.52**	**2.07 (1.51, 2.83)**
3	IS & CS	PI	15.72	75.04	1.01	1.26 (1.09, 1.47)
3	CS & BS	PI	7.17	74.63	1.00	1.28 (1.04, 1.58)
4	**IS & CS & BS**	**PI**	**3.59**	**76.89**	**1.03**	**1.41 (1.05, 1.89)**
4	OB & CS & BS	PI	2.86	75.35	1.01	1.32 (0.96, 1.82)

Rules are listed by order of predicted variables and support values in descendent order.

a ARM results of Minimum support 2%; minimum confidence: 60% for men, 50% for women.

b Size is the number of lifestyle risk behaviors included in the rule. Predictors are the variables to the left of the rule, and predicted variables are those to the right of the rule.

c The odds ratio was adjusted for sex, age, marital status, education, occupation, income, and chronic disease status.

CS: current smoking; HD: heavy drinking; PI: physical inactivity; OB: obesity; IS: inadequate sleep; BS: breakfast skipping; FS: frequent snacking.

Four association rules met our threshold among all participants. According to these rules, individuals with PI and HD together were more likely to be CS. The confidence level of 56.41% means that 56.41% of individuals who were both physically inactive and heavy drinkers were current smokers. The support measure of 3.13% indicates that for the whole study sample, 3.13% reported simultaneous physical inactivity, heavy drinking, and current smoking. The lift value of 2.64 shows that the ratio of the proportion of participants who were current smokers (among individuals reporting PI and HD) to the proportion of participants who were current smokers (in the whole sample) was 2.64. In other words, the probability of being a current smoker while *simultaneously* being physically inactive and drinking heavily was 2.64 times higher than the probability of simply being a current smoker. Therefore, physical inactivity and heavy drinking *together* were positively associated with current smoking.

For men, there were 10 association rules involving current smoking and physical inactivity. According to these rules, individuals who were both heavy drinkers and breakfast skippers were more likely to be current smokers. Individuals who reported the three simultaneous risk behaviors of inadequate sleep, current smoking, and breakfast skipping were more likely to be physically inactive. Breakfast skippers with additional risk behaviors such as physical inactivity, obesity, and inadequate sleep were more likely to be current smokers. Furthermore, current smokers with additional risk behaviors such as inadequate sleep and breakfast skipping were also more likely to be physically inactive.

There were fewer association rules for women than for men; therefore, we set the confidence threshold at 50%, 10% lower than that for the analysis of the male participants’ data. We found 5 association rules that met this new threshold; however, the rules were somewhat different for women than for men, and primarily implicated inadequate sleep. According to these rules, women who were current smokers were more likely to report inadequate sleep. Women who were obese and skipped breakfast were also more likely to have inadequate sleep.

We observed several gender differences in the patterns of risk behaviors between the male and female participants. For the men, patterns mainly involved CS and PI, but among the women, patterns mainly involved IS: the female obese breakfast skippers were more likely to report inadequate sleep (lift 1.02, odds ratio 1.27, *p = *0.0297) while the male obese breakfast skippers were more likely to be current smokers (lift 1.42, odds ratio 1.77, *p*<0.0001).

This pattern of the simultaneous occurrence of PI, CS, and IS was observed for both women and men. Among women, co-occurrence of PI and CS was a predictor of IS, while among men, co-occurrence of IS and CS was a predictor of PI. The cluster of behaviors was the same, but the direction was different.

**Figure 3:**
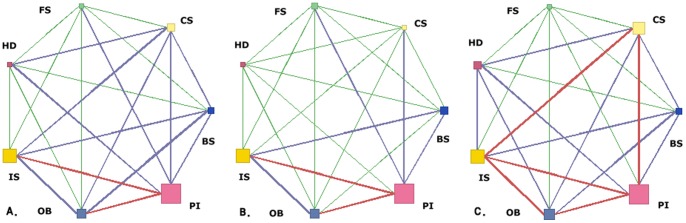
A link diagram derived from association rule mining (ARM). Link Analysis of SAS Enterprise Miner. Results of A. overall, B. women, and C. men participants. The size of each node indicates the frequency of the behavior it represents. The color of each line indicates the frequency of the link it represents. Red links have the highest, blue the middle, and green the lowest frequency.

Figure 3 shows a network of associations and illustrates their strengths and frequencies. For the whole sample (Figure 3A.), PI, IS, and OB nodes were the largest, and the lines linking PI with both IS and OB were most prominent. Among women (Figure 3B.), PI, IS, and OB nodes were the largest, and the 2 lines linking the PI and IS nodes and the PI and OB nodes were most prominent. Among men (Figure 3C.), PI, IS, CS, and OB nodes were largest, and the five lines linking these nodes were the most prominent. This illustrates that men were more likely to engage in multiple lifestyle risk behaviors than women.

### Factors Predicting Lifestyle Risk Behavior Patterns

We selected rules with higher lift value and significant odds ratios, and considered individuals whose behaviors matched these rules to form a multiple lifestyle risk group. We conducted a multiple logistic regression analysis to predict important variables for the multiple lifestyle risk group (Table 3). Among women, young age, marital status (separated, divorced, widowed, or never married), lower education, lower income level, and higher perceived stress were all predictors of the combination of current smoking and insufficient sleep. Among men, young age, marital status (separated, divorced, or widowed), higher perceived stress, and low self-rated health status were all predictors of the combination of being a heavy drinker, a breakfast skipper, and a current smoker. In addition, among men, young age, marital status (separated, divorced, widowed, or never married), lower income level, and higher perceived stress status were all predictors of the combination of insufficient sleep, current smoking, breakfast skipping, and physical inactivity.

**Table 3:** Multiple logistic regression analysis for predicting patterns of multiple lifestyle risk behaviors.

	Model		
	Women CS & IS^a^	Men HD, BS & CS^b^	Men IS, CS, BS & PI^c^
Age			
20–44	1	1	1
45–64	0.3 (0.2, 0.5)	0.4 (0.2, 0.7)	0.5 (0.4, 0.8)
65+	0.3 (0.1, 0.5)	0.0 (0.0, 0.1)	0.1 (0.0, 0.2)
Marital status			
Married	1	1	1
Separated/divorced/widowed	2.6 (1.8, 3.9)	2.0 (1.0, 4.0)	1.8 (1.0, 3.4)
Never married	2.4 (1.5, 3.7)	1.2 (0.7, 2.0)	1.8 (1.2, 2.7)
Education			
College	1	1	1
High school	2.5 (1.5, 4.3)	1.3 (0.7, 2.2)	1.0 (0.7, 1.5)
Middle school	4.7 (2.2, 10.2)	1.6 (0.7, 3.9)	1.1 (0.6, 2.2)
None or elementary school	4.6 (2.0, 10.6)	1.2 (0.5, 3.4)	0.9 (0.4, 2.0)
Occupation			
Office worker	1	1	1
Manual worker	1.4 (0.9, 2.3)	1.0 (0.6, 1.7)	0.8 (0.5, 1.2)
Unemployed	1.2 (0.8, 1.9)	0.7 (0.4, 1.3)	0.6 (0.3, 1.1)
Income			
1st quartile	1	1	1
2nd quartile	1.0 (0.7, 1.6)	1.5 (0.9, 2.6)	1.2 (0.8, 1.9)
3rd quartile	1.5 (1.0, 2.3)	1.5 (0.8, 2.7)	1.4 (0.9, 2.2)
4th quartile	1.5 (0.9, 2.3)	1.3 (0.6, 2.7)	1.8 (1.1, 3.2)
Perceived stress			
No	1	1	1
Yes	2.1 (1.6, 2.9)	1.5 (1.0, 2.2)	1.8 (1.3, 2.5)
Self rated health			
Good/Fair	1	1	1
Bad	1.1 (0.8, 1.6)	1.9 (1.2, 3.1)	1.1 (0.7, 1.7)
Chronic disease			
No	1	1	1
Yes	1.4 (0.9, 2.1)	1.1 (0.7, 1.6)	0.8 (0.6, 1.1)

Logistic regression models were constructed with survey logistic procedure using survey sample weights.

a Model for Women CS & IS: the model predicting the probability of simultaneous CS & IS.

b Model for Men HD, BS & CS: the model predicting the probability simultaneous HD, BS & CS.

c Model for Men IS, CS, BS & PI: the model predicting the probability of simultaneous IS, CS, BS & PI.

CS: current smoking; HD: heavy drinking; PI: physical inactivity; OB: obesity; IS: inadequate sleep; BS: breakfast skipping; FS: frequent snacking.

## Discussion

Our study demonstrated that the most frequently occurring lifestyle risk behaviors among Korean adults were physical inactivity and insufficient sleep. The ARM analysis revealed patterns of risk behaviors involving current smoking, insufficient sleep, and physical inactivity.

### Most Prevalent Lifestyle Risk Behaviors

Among the risk behaviors, the overall prevalence rates of physical inactivity and insufficient sleep were the highest. Similar results were observed in a previous study [30] showing that approximately 51% of American adults do not meet the recommended guidelines for physical activity according to Behavioral Risk Factor Surveillance System (BRFSS) data. Inadequate sleep was also a common factor in the BRFSS data. Strine and colleagues revealed that an estimated 26% of adults reported frequent sleep insufficiency [31].

### Patterns of Lifestyle Risk Behaviors

Our study adds to the growing body of research exploring the association between multiple lifestyle risk behaviors. Studies exploring the associations between 3 to 5 risk behaviors have demonstrated that most people exhibit either 1–2 risk behaviors [8] or 2–3 risk behaviors [12]. Our study expanded on these findings by exploring the co-occurrence of 7 lifestyle risk behaviors and finding meaningful association rules for combinations of 2 to 5 behaviors. Our findings also demonstrated sex differences in lifestyle risk behaviors and showed that multiple risk behavior patterns are more frequent in men than in women. These results confirm previous analyses of health behaviors according to sex [6], [7], [8], [9], [32].

Men who were physically inactive, heavy drinkers, breakfast skippers, and obese were more likely to be current smokers. Previous studies have found that current smoking can be either positively [6], [32] or negatively [8], [12] associated with physical inactivity. This inconsistency in results may be related to differences in participant age groups between studies. Alternatively, it is possible that the association reflects the finding that people in manual occupations are more likely to smoke [12]. Researchers have suggested that, among current smokers, exercise reduces smoking by reducing the urge to smoke [35], and that physical inactivity is positively associated with breakfast skipping [34]. Current smoking is also positively associated with heavy drinking [6], [8], [12], [32]; however, the present study found that the probability of current smoking was associated with not only heavy drinking but also physical inactivity, obesity, and breakfast skipping. Our results were consistent with a previous finding that breakfast skipping is moderately clustered with smoking, alcohol use, and a sedentary lifestyle [33], and were similar to those of Sakata et al. [34], who found that breakfast skippers tend to smoke more than did non-skippers [34]. Together, these findings confirm our rule showing that physical inactivity was associated with a combination of current smoking, and breakfast skipping.

Among men, inadequate sleep with additional risk behaviors was associated with current smoking and physical inactivity. However, a different pattern emerged for women in which current smoking, physical inactivity, and obesity were associated with insufficient sleep. These findings are similar to those of another study [31] that found a relationship between insufficient sleep and smoking, physical inactivity, and obesity. Previous research has also reported an effect of insufficient sleep on obesity such that sleep-restricted participants reported a higher average body mass index [36] compared with adequate sleepers.

### Factors Predicting Lifestyle Risk Behavior Patterns

In the present study, the logistic regression analysis showed that multiple lifestyle risk behaviors are associated with sex (males), age (younger age groups), marital status (separated, divorced, or widowed), and education (lower levels). This is consistent with a growing body of research on lifestyle risk behaviors [7], [12], [32], and indicates the need for worldwide public health initiatives focused on modifying these behaviors.

A number of variables contribute to the probability of lifestyle risk behaviors. For example, in the current study, perceived stress was important for predicting multiple lifestyle risk behaviors while chronic disease status was not. This is consistent with the finding that the probability of multiple lifestyle risk behavior increases when mental distress is high [7]. However, this finding is somewhat controversial because previous studies have demonstrated that chronic disease status [7] or better perceived health status [6] increases the probability of exhibiting multiple lifestyle risk behaviors. A possible explanation for this discrepancy is the behavior patterns that included current smoking with additional behaviors of inadequate sleep, breakfast skipping, heavy drinking, and physical inactivity. Thus, the present study contributes to the understanding of the interrelation among specific lifestyle risk behaviors, and has identified some that may be of greatest consequence.

### Study Strengths

The current study has important implications for interventions to modify lifestyle risk behaviors. We found that many individuals practice a number of risk behaviors simultaneously, and identified specific association rules between these behaviors using ARM. Previous studies have used different analytical techniques to identify associations between lifestyle risk behaviors. For example, researchers have measured associations by using behavior accumulation techniques [32], the prevalence of odds ratios [9], [12], correlation analysis [37], cluster analysis [38], and various regression approaches [5], [32].

Using accumulation technique methods, one cannot detect specific behavior combinations. In order to study the association structure of 7 binary health risk behaviors, we would need to analyze a contingency table with 2×2^7^ possible levels. Consequently, there will be a number of empty cells; an exhaustive analysis of the table is challenging. With correlation analysis, regression approaches, and odds ratios, behavioral associations are generally studied from the perspective of a single behavior with preconceived ideas about the order of importance of behaviors. This can lead researchers to overemphasize the role of the primary selected behavior [32]. The present study avoided this problem by utilizing ARM, a technique that assumes no hierarchy of lifestyle risk behaviors and creates simple association rules between three or more behaviors.

Although lifestyle risk behavior patterns can be obtained by logistic regression analysis, ARM offers these patterns in the form of rules with support, confidence, and lift, according to desired thresholds for support, confidence, and lift. Most importantly, this approach allowed us to investigate relevant patterns and to determine the direction of associations between behaviors. This approach enabled us to predict other risk behaviors in which individuals might engage.

In addition, a particular strength of our study is that we analyzed a large, representative sample of the general adult population of Korea, using comprehensive lifestyle behavior questionnaires. Finally, our analysis of lifestyle risk behaviors was conducted according to the Alameda 7 healthy behaviors, which together indicate healthy lifestyle choices.

### Limitations

The present study has some important limitations. First, we analyzed cross-sectional data; therefore, it is not possible to infer causal relationships. However, the association rules can be used to estimate the probability of related behaviors. Second, we considered lifestyle risk behaviors according to public health recommendations, and our findings were affected by these criteria. Third, we could not explain sex differences in the associations of risk behavior patterns; further studies are needed to determine the factors associated with these differences.

### Interventions Targeted at Multiple Lifestyle Risk Behaviors

Interventions to reduce multiple risk behaviors simultaneously are important because people with lifestyle risk behaviors that remain unchanged have worse self-reported health status than those who are able to make lifestyle changes [5]. For example, the modification of multiple lifestyle risk behaviors could potentially reduce the risk of acute myocardial infarction (heart disease) by more than three-quarters [3]. Combining smoking and weight control intervention programs may reduce the relapse of risk behaviors [39].

The ARM results reported here may also be useful in the field of health promotion. For example, if smokers are intervention targets, instead of focusing solely on smoking, programs could incorporate exercise, regular eating habits, and alcohol prevention. Applying ARM results in health promotion programs should help prevent risk behaviors and thus reduce the risk of chronic disease.

### Conclusion

Lifestyle risk behaviors tend to be intercorrelated. Inadequate sleep, current smoking, and physical inactivity are associated with additional risk behaviors. Our study demonstrates several possible points of intervention. To modify the behavior of women with insufficient sleep, preventative programs should take into account associated risk behaviors, namely, smoking habits, obesity, and breakfast skipping. To modify the behavior of male smokers, prevention programs should take into account breakfast skipping, physical inactivity, inadequate sleep, and obesity. To modify the behavior of physically inactive men, programs should address smoking habits, sleep habits, and breakfast skipping. Thus, information on the patterns of lifestyle risk behaviors should support the development of more effective multiple-behavior intervention programs.
